# Deep Learning-Based Decoding and Feature Visualization of Motor Imagery Speeds From EEG Signals

**DOI:** 10.1109/OJEMB.2025.3645617

**Published:** 2025-12-18

**Authors:** Shogo Todoroki, Chatrin Phunruangsakao, Keisuke Goto, Kyo Kutsuzawa, Dai Owaki, Mitsuhiro Hayashibe

**Affiliations:** ^1^ Department of Robotics, Graduate School of EngineeringTohoku University Sendai 980-8579 Japan; ^2^ Neuro-Robotics Laboratory, Graduate School of Biomedical EngineeringTohoku University Sendai 980-8579 Japan

**Keywords:** Brain-computer interface, deep learning, explainable artificial intelligence, motor imagery, speed decoding

## Abstract

*Objective:* This study investigates the neurodynamics of motor imagery speed decoding using deep learning. *Methods:* The EEGConformer model was employed to analyze EEG signals and decode different speeds of imagined movements. Explainable artificial intelligence techniques were used to identify the temporal and spatial patterns within the EEG data related to imagined speeds, focusing on the role of specific frequency bands and cortical regions. *Results:* The model successfully decoded and extracted EEG patterns associated with different motor imagery speeds; however, the classification accuracy was limited and high only for a few participants. The analysis highlighted the importance of alpha and beta oscillations and identified key cortical areas, including the frontal, motor, and occipital cortices, in speed decoding. Additionally, repeated motor imagery elicited steady-state movement-related potentials at the fundamental frequency, with the strongest responses observed at the second harmonic. *Conclusions:* Motor imagery speed is decodable, though classification performance remains limited. The results highlight the involvement of specific frequency bands and cortical regions, as well as steady-state responses, in encoding MI speed.

## I. Introduction

Brain-computer interfaces (BCIs) enable direct communication between the brain and external devices, offering a promising approach to assist individuals with motor impairments by allowing control over prosthetic systems through brain activity [1]. Among these, motor imagery (MI)-based BCIs decode neural signals generated during the mental rehearsal of movement, enabling interaction without physical execution and supporting neuroplasticity [2], [3]. Electroencephalography (EEG) is widely used in MI-BCIs due to its high temporal resolution and cost-effectiveness, although its limited spatial resolution makes it difficult to distinguish tasks involving overlapping motor areas [4], [5].

Despite more than two decades of pioneering research, the clinical adoption of MI-BCIs remains limited. Most studies have been confined to laboratory settings, and few systems have demonstrated consistent clinical benefits or gained widespread acceptance among rehabilitation specialists [5], [6]. Nonetheless, MI-BCIs hold strong potential as tools for neurorehabilitation. Several studies have employed deep learning approaches to classify various MI tasks, including single-limb [7], [8], bimanual [6], [9], [10], and continuous tracking tasks [11]. Although these models often achieve high decoding accuracy, their decision-making processes remain opaque, limiting understanding of the learned neural dynamics and the opportunity to design improved models with higher accuracy. With the rise of explainable artificial intelligence (xAI), these techniques can be incorporated to reveal what the models learn and how these representations relate to brain activity, ultimately guiding the design of more interpretable and effective MI-BCI systems for clinical use and real-world applications.

Decoding movement speed—a parameter reflecting the frequency of repetitive imagined motions—has been relatively overlooked. Most existing MI-BCI systems operate at a single, fixed speed, whereas everyday movements naturally vary in pace depending on the task and context [1], [5]. Accurate decoding of movement speed is therefore critical for MI-BCI–based training, as it enables real-time adjustment of task difficulty, supports graded neurofeedback, and promotes neuroplasticity, all of which are key for optimizing motor learning and rehabilitation outcomes. Furthermore, precise speed decoding allows MI-BCI systems to provide smooth and naturalistic control, which is essential for translating laboratory-based interventions to functional real-world tasks such as walking, reaching, or object manipulation.

Studies investigating neural correlates of imagined speed have yielded mixed results. Yuan et al. [12] identified a linear correlation between alpha and beta frequency band activity and the speed of MI clenching. Additionally, studies by Fu et al. [13], [14] noted that slow MI tasks exhibit larger positive slow potentials ($< $2 Hz) compared to faster tasks. However, Ding et al. [15] investigated EEG activity under varying speeds and task prompts, revealing no linear correlations between motion speeds and changes in alpha and beta bands. Despite these contrasting findings, several studies have attempted to decode speed variations in tasks such as hand clenching [16], finger tapping [17], and arm movements [18], [19], [20] from event-related (de)synchronization (ERD/S) signals. A study by Wei et al. [21] utilized steady-state movement-related potentials (SSMRP) to decode movement speed, achieving high accuracy. They discovered that both the accurate movement speed and its first harmonic were encoded in the contralateral motor cortex. However, the scope of previous research remains limited by the number of classes involving different speeds and the specific body parts performing the movements, with some studies reporting contrasting findings.

Deep learning has advanced MI-BCI by enabling automatic extraction of spatial, spectral, and temporal features [22]. Convolutional and recurrent neural networks (CNNs and RNNs) have shown effectiveness in MI classification [23], [24], [25], [26], [27], though CNNs may struggle to capture long-term dependencies, and RNNs are prone to gradient vanishing issues. Recently, transformer-based models such as EEGConformer [28] have demonstrated superior performance by capturing global temporal patterns. Despite their accuracy, deep models often lack interpretability [29]. xAI techniques help address this by making model decisions more transparent and offering insights into the neural processes involved in MI. Tools like Grad-CAM [30] applied to transformer-based models enable visualization of important spatiotemporal features [28].

This study employs EEGConformer to classify imagined grasping movements performed at different speeds using EEG, leveraging its state-of-the-art performance. Fourier analysis and mixing matrices are applied to identify key frequency and spatial features from the convolution module of EEGConformer. Furthermore, two feature visualization techniques, Class Activation Mapping (CAM) and Class Activation Topography (CAT), are employed to observe the global representations learned by the transformer modules for each MI task. CAM and CAT are utilized to visualize temporal and spatial attention to the EEG features, respectively.

## II. Results

### A. Classification Performance

The performance metrics include accuracy, F1-score, and Cohen's kappa coefficient ($\kappa$). The classification performance is summarized in Table 1. The mean accuracy achieved was 61.97% ($SD = 10.10\%$), with a corresponding mean F1-score of. 5698 ($SD =. 1064$) and a mean $\kappa$ of. 4621 ($SD =. 1435$). The results demonstrate the feasibility of decoding MI speed across participants, although performance varied considerably. While some participants exhibited relatively high accuracy, others showed lower performance, reflecting inter-subject variability and individual differences in neural patterns and engagement during the MI task, and highlighting the need for further optimization and validation in larger and more diverse cohorts to achieve performance suitable for real-world applications.

**TABLE 1:** Comparison of Classification Performance Across Participants. The Classification Chance Level is 0.2857. $M$ Denotes the Mean, $SD$ Represents the Standard Deviation, and $\kappa$ Indicates Cohen's Kappa Coefficient.

Participant	Accuracy	F1-score	$\kappa$
1	63.33	. 5540	. 4762
2	50.00	. 4453	. 2987
3	67.78	. 6545	. 5469
4	65.56	. 6188	. 5194
5	54.44	. 5103	. 3577
6	51.11	. 4211	. 2903
7	80.00	. 7545	. 7143
8	47.78	. 2637	. 2637
9	65.56	. 5194	. 5194
10	77.78	. 6867	. 6867
11	62.22	. 4560	. 4560
12	54.44	. 3627	. 3627
13	65.56	. 5156	. 5156
$M$	61.97	. 5698	. 4621
$SD$	10.10	. 1064	. 1435

**Figure 1.**
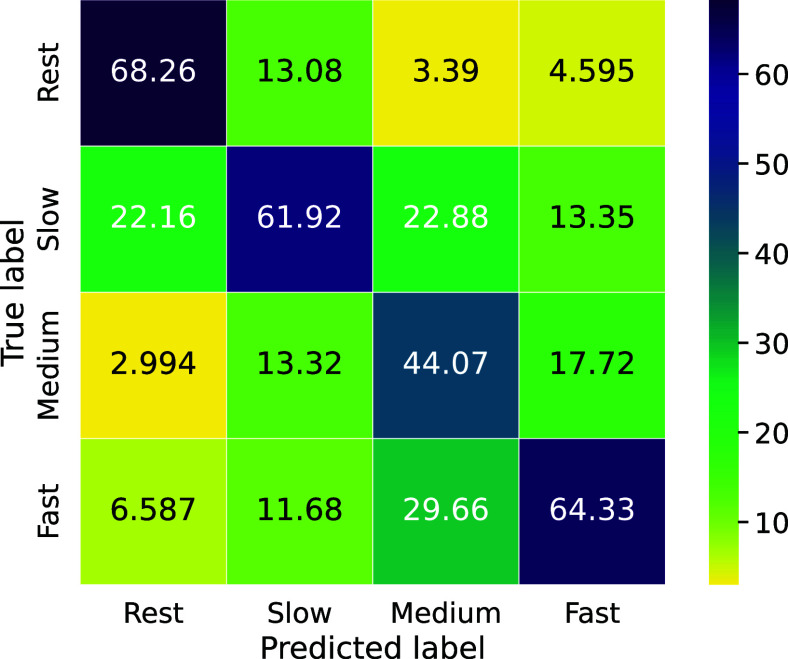
Confusion matrix illustrating the classification performance of the model in distinguishing between four motor imagery tasks. The matrix displays the percentage of trials (y-axis) predicted for each class (x-axis), highlighting a high confusion rate, especially for the intermediate speeds.

Fig. 1 presents the confusion matrix normalized by predicted labels, and Table 2 summarizes the corresponding precision and recall. Overall, the *rest* task is the most accurately classified across the four conditions. In contrast, the classification of grasping speeds shows more confusion, particularly when the speeds are close to each other. Specifically, *slow* is often misclassified as *rest*, *medium* is confused with both *slow* and *fast*, and *fast* is frequently misclassified as *medium*. This pattern suggests that the model struggles to differentiate between adjacent MI speeds.

**TABLE 2:** Precision and Recall Scores for Each Motor Imagery Task. $M_{\textit{weighted}}$ Represents the Weighted Mean Across All Classes.

Task	Precision	Recall
Rest	. 6826	. 5846
Slow	. 6192	. 6795
Medium	. 4407	. 2667
Fast	. 6433	. 7538
$M_{weighted}$	. 5964	. 5712

### B. Steady-State Movement-Related Potential

**Figure 2.**
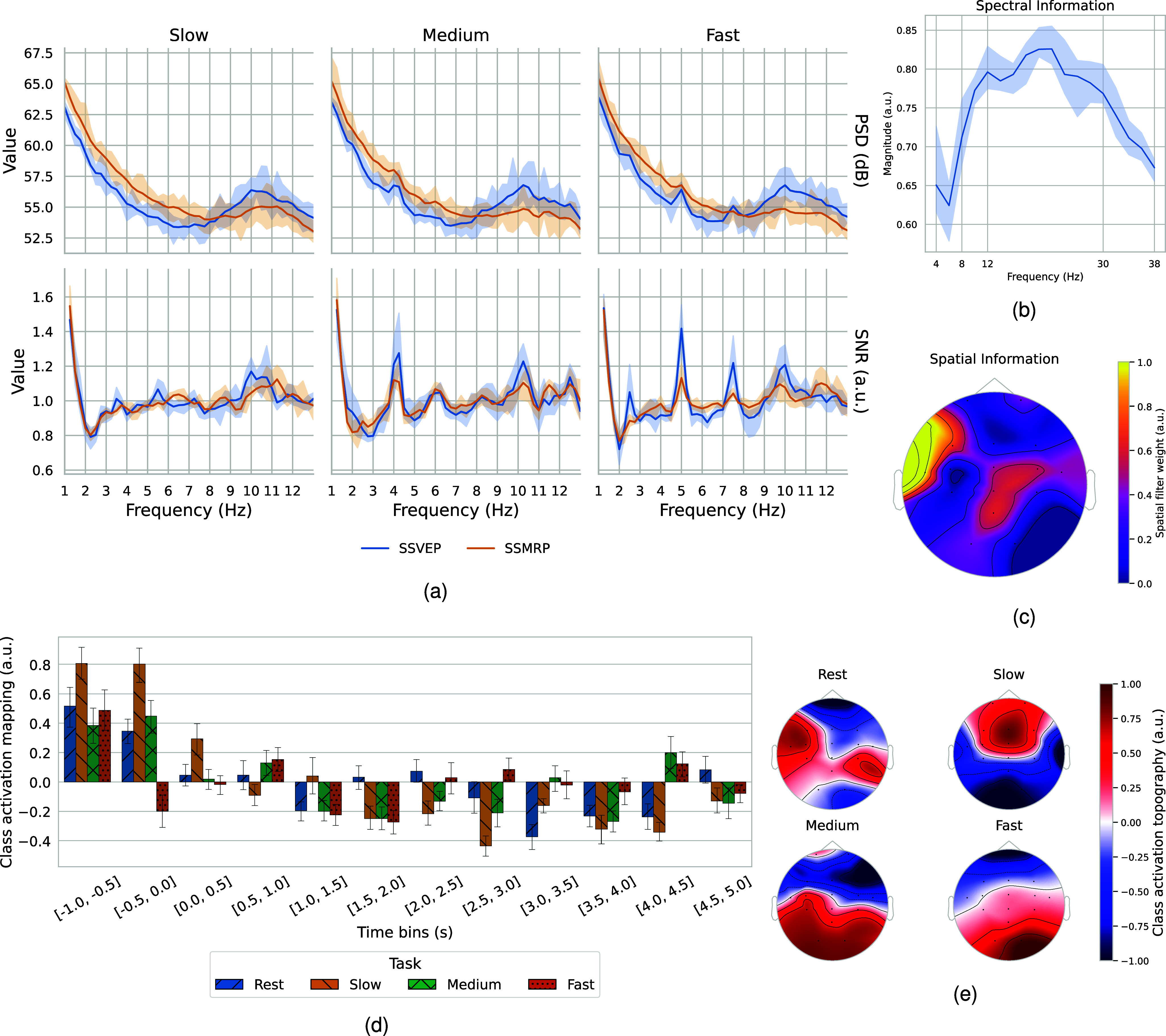
Feature visualization. (a) PSD and SNR plots for each MI speed. Distinct peaks appear at the frequencies corresponding to the *medium* (2.1 Hz) and *fast* (2.6 Hz) conditions and their harmonics, whereas the *slow* condition shows weaker spectral peaks. PSD was computed using Welch's method with a Blackman window and converted to dB. SNR was calculated as the power ratio between each frequency and the mean of its $\pm$5 neighboring bins, excluding edge frequencies. (b) The frequency response of the temporal convolution filters shows high magnitudes in the alpha and beta bands. (c) The spatial pattern exhibited notably high weights in the motor cortex and the right temporal lobe. (d) The CAM plot highlights the time periods most attended by the model. Positive CAM values indicate ERS, while negative values indicate ERD used for class discrimination. The results show strong attention during pre-movement and attenuated attention during movement. (e) The CAT plot is generated by multiplying the normalized EEG signals with the CAM, visualizing the spatial regions where the model directs its attention, and showing elevated focus on the frontal, motor, and occipital areas.

The averaged PSD and SNR plots across participants, as depicted in Fig. 2(a), reveal the spectral signatures of both SSVEP and SSMRP, most prominently in the *medium* and *fast* conditions, while the *slow* condition shows no noticeable response. Distinct peaks are present at the fundamental frequencies—approximately 2.1 Hz for *medium* and 2.6 Hz for *fast*—with the highest magnitudes appearing at the second harmonic (i.e., 4.2 Hz for *medium* and 5.2 Hz for *fast*), followed by a gradual attenuation in power at higher harmonics. Notably, the magnitude of the SSVEP response consistently exceeded that of the SSMRP. All in all, these results confirm that both SSVEPs and SSMRPs are observable when participants perform repetitive MI tasks under visual stimulation.

### C. Frequency Response and Spatial Pattern

The average frequency response and spatial pattern of the filters in the convolution module are depicted in Fig. 2(b) and (c), respectively.

The magnitude of the frequency response is lowest in the theta band (4–8 Hz), increases in the alpha band (8–12 Hz), and peaks in the beta band (12–30 Hz) with a gradual decrease in magnitude in higher frequencies. These patterns indicate that the temporal filters were optimized to capture the neural oscillations relevant to MI speed, particularly those in the alpha and beta bands, which are associated with motor planning and execution.

Spatial filter weights are visualized as normalized absolute values, as their sign contains no information about oscillatory activity [31]. High spatial weights are observed in the left temporal channel and motor-related regions, including channels Cz, C4, T7, CPz, and Pz.

### D. Global Representation

The temporal dynamics of model attention across time bins for each MI task are represented by the CAM values in Fig. 2(d). CAM values can be noisy and difficult to interpret at the original time resolution; therefore, to reduce this variability and enhance interpretability, the CAM was visualized using time bins of 0.5 seconds with a step size of 0.5 seconds. Notably, distinct activation patterns emerge across different MI speeds, particularly during the task period. During the pre-task period (-1 to 0 s), CAM values are elevated, suggesting anticipatory neural patterns that the model identifies as discriminative. Moreover, during the MI period, the CAM values show attenuation, which may correspond to the ERD phenomenon associated with motor imagery.

The model also uncovered distinct spatial attention patterns across MI speeds, as illustrated in Fig. 2(e). Specifically, the *medium* and *fast* conditions show high attention in occipital regions and attenuated attention particularly in frontal regions, whereas the *slow* condition exhibits the opposite trend, with high frontal and low occipital attention. The *rest* condition shows a more distributed spatial attention pattern.

## III. Discussion

The extent to which visual stimulation and repetitive rhythmic movement can elicit steady-state potentials, such as SSVEP and SSMRP, remains an open question. Wei et al. [21] reported that varying movement speeds evoke SSMRP at corresponding fundamental frequencies and harmonics, particularly in the contralateral motor cortex. Consistent with this, the PSD and SNR plots in Fig. 2(a) confirm the presence of both SSVEP and SSMRP during repetitive motor imagery combined with visual stimulation. However, these components were not observed under the slow condition, likely due to weaker neural responses and lower SNR. The low-frequency SSVEP band (1–12 Hz), which includes the stimulation frequencies used in this study, is known to elicit weaker responses compared to the mid-frequency range (12–30 Hz) [32]. Additionally, the nature of the visual stimuli in this experiment may have contributed to the reduced SSVEP strength. These factors likely account for the absence of detectable steady-state components in the *slow* condition.

The temporal filters of the convolution module optimized the bandpass frequency within the alpha and beta bands, as depicted in Fig. 2(b). Motor programming is closely linked to alpha-like oscillations in the sensorimotor cortex, known as the mu rhythm, which plays a crucial role in regulating cortical excitability. Mu oscillations exhibit ERD in task-relevant regions and ERS in task-irrelevant regions [33]. The beta band is also associated with sensorimotor processing, making frequency extraction within the alpha and beta bands particularly valuable for highlighting the sensory, cognitive, and motor processes necessary for decoding MI speeds [34], [35], [36], [37]. Furthermore, studies [12], [16], [38] have shown that relative changes in both alpha and beta bands are linearly correlated with the speed of both MI and the motor execution of hand movements.

The spatial convolution layer revealed a strong focus on the motor cortex, parietal cortex, and right temporal area, as evident in Fig. 2(c). Mirror neurons, which are activated during both the execution and observation of movement, are closely associated with the mu rhythm [39], [40]. These neurons are consistently found in the motor cortex [41]. Observing hand actions primarily activates the lateral premotor cortex, posterior temporal cortex, and extrastriate visual cortex, with minimal consistent activation in the parietal areas [42]. During MI, kinematic parameters such as movement speeds are encoded in the motor cortex [12], [16]. Additionally, the temporal lobe is linked to global visual processing and the encoding and retrieval of memories [43], [44]. A study by Breault et al. [45] also reported that features in the right hippocampus, left and right middle temporal gyrus, intraparietal sulcus, and left fusiform gyrus play a significant role in decoding speeds. Taken together, these findings suggest that the observed activations in motor regions may reflect a combination of motor imagery–related processes, including the encoding of movement parameters, and potential mirror neuron activity elicited by the observation of hand movement videos used as pacing cues. Meanwhile, temporal activations may correspond to visual information processing, attentional modulation, and memory-related functions. Future investigations should aim to dissociate the respective contributions of motor imagery and visual observation by employing alternative stimulus modalities or including motor execution conditions.

The CAM plot, as demonstrated in Fig. 2(d), revealed that the model paid particularly high attention to the input signals prior to movement onset. Modulations within the alpha and beta bands, especially ERD, have been shown to occur as early as 2 seconds before voluntary movements [46], [47]. Additionally, ERD during both pre-movement and movement periods has been demonstrated to encode peak speed and acceleration, particularly in the occipital and parietal–occipital cortices for the alpha band, and in the frontal and frontal-central lobes for the beta band [48]. Therefore, pre-movement ERD could reflect efficient movement planning. The model also appears to utilize information from approximately between 1.0 to 4.0 seconds after movement onset. Several studies on time window optimization for EEG decoding have reported similar findings, indicating that this window yields the highest classification accuracy [49], [50].

While the present study filtered out the low-frequency components of EEG signals during preprocessing to avoid motion artifacts, this approach may also eliminate the movement-related cortical potential (MRCP), a low-frequency ($< $4 Hz) negative potential that occurs before movement onset. The maximum negative offset of the MRCP has been shown to correlate with the amount of torque required to execute a movement, with higher torque tasks resulting in a larger negative offset [6], [51], [52]. Given that torque level and movement speed are mechanically related, the MRCP may also encode specific parameters related to speed. Therefore, further research should reconsider the inclusion of these low-frequency components.

The CAT plot reveals a pronounced concentration of activity in the frontal and occipital lobes, with moderate attention to motor-related regions, as reflected in Fig. 2(e). Although the precise nature of the activity remains unspecified, these regions are likely crucial for the accurate classification of MI speeds. Within the frontoparietal network, frontal alpha oscillations are believed to reflect top-down control mechanisms that modulate perceptual gains, thereby affecting parietal mu oscillations associated with multisensory integration [53]. Additionally, the frontal cortex may contribute to the precise timing necessary for executing MI at different speeds. Temporal information is decoded in both the parietal and frontal cortices, with time representation being widely distributed across frontoparietal regions. Specifically, neural populations in the right parietal cortex play a significant role in time estimation [54], [55]. The activation of the frontal, parietal, and occipital regions may facilitate visuomotor integration, converting visual information into movement kinematics and supporting action planning during visual stimulation [56], [57], [58].

Although this study provides valuable insights into the neurodynamics associated with MI speeds, several limitations should be acknowledged. The reliability of Grad-CAM, depends on model accuracy, which may be constrained by the relatively small training dataset. In this study, small trainng data were used to train the model, increasing the risk of overfitting and potentially limiting its performance. Additionally, Grad-CAM's temporal and spatial resolution may be insufficient to capture fine-grained neural dynamics. The participant group was also limited to a small number of right-handed individuals within a narrow age range, which may not adequately represent the general population. Consequently, the observed findings may only be applicable to a specific demographic group. Furthermore, the results exhibited large inter-subject variability in decoding performance, which could lead to less reliable interpretations from the xAI analysis. This variability may also be attributed to the well-known issue of BCI illiteracy, where certain individuals are unable to generate sufficiently discriminable neural patterns during MI. Future work should therefore involve a larger and more diverse participant population, and consider strategies such as data augmentation or transfer learning to improve model robustness with limited data, as well as employ higher-resolution xAI methods for more precise and generalizable interpretations.

## IV. Conclusion

This study demonstrates that EEGConformer can decode motor imagery speed by capturing relevant temporal and spatial EEG patterns, particularly in alpha and beta bands and sensorimotor regions. Temporal attention suggests the model adaptively focuses on informative periods. However, performance varied across participants and was limited by the small dataset, model complexity, resolution constraints of xAI, and a narrow participant group. Future work should expand the dataset and population, apply methods such as data augmentation or transfer learning, and incorporate higher-resolution explainability tools to enhance model reliability, generalizability, and clinical relevance.

## V. Materials and Methods

### A. Participants

Thirteen right-handed participants ($M_{age}=23.46$, $SD_{age}=1.65$, 10 males, 3 females) with no known neurological conditions, and normal or corrected-to-normal eyesight were recruited in this study.

### B. Experimental Design and Protocol

**Figure 3.**
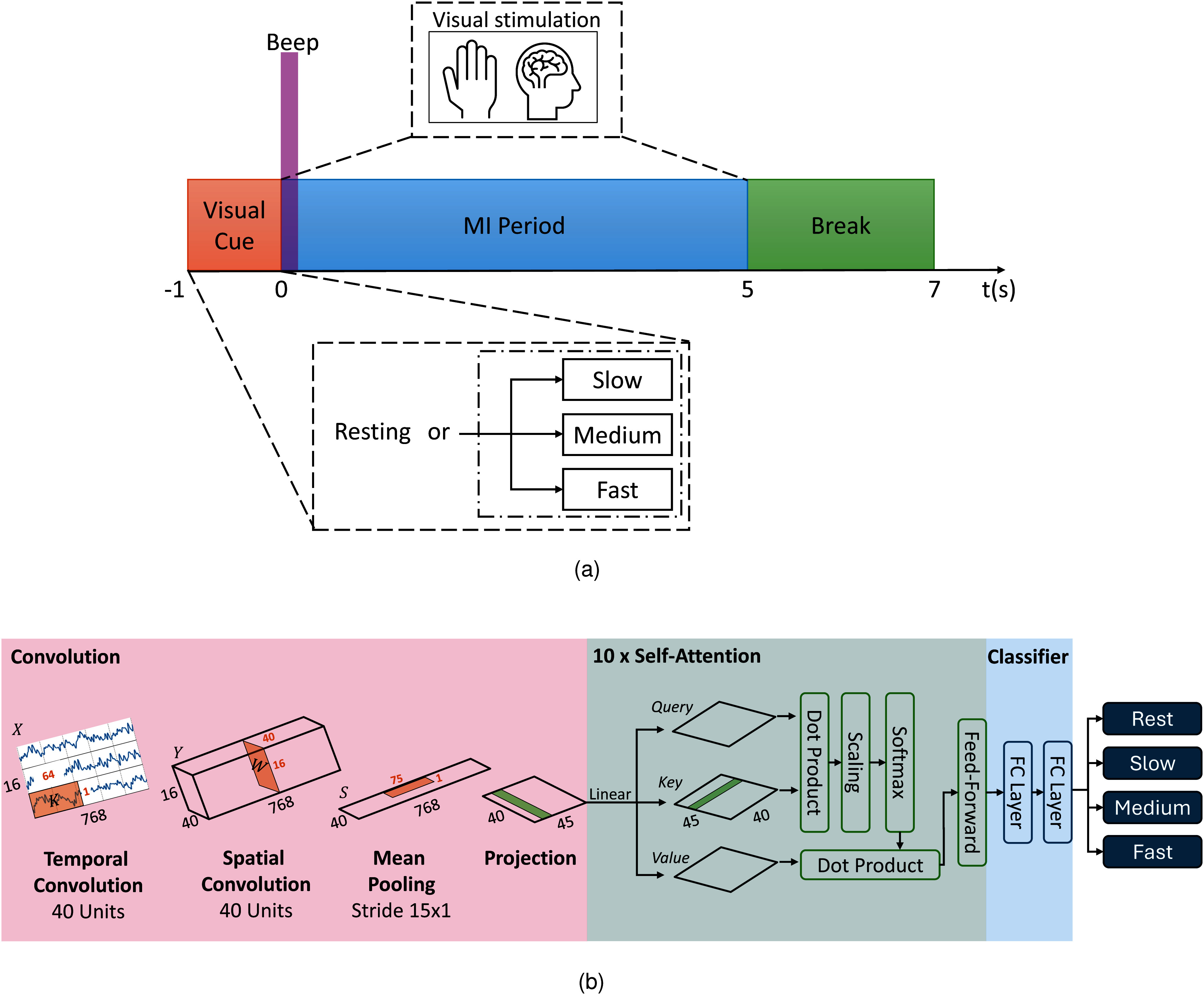
Overview of the experimental design: (a) Experimental timing - each trial follows this sequence: visual cue, MI period, and a brief break. (b) EEGConformer network architecture: The model consists of three modules - convolution, self-attention, and classification [28].

The EEG recording followed the timing scheme depicted in Fig. 3(a). Each trial began with a 1-s visual cue indicating the MI task to be performed. The tasks consisted of imagined grasping movements using either left or right hand at approximate speeds of 0.93 Hz, 2.12 Hz, and 2.56 Hz, corresponding to the *slow*, *medium*, and *fast* conditions, respectively, along with a *rest* condition. These frequencies were selected to ensure perceptually distinct pacing rates while avoiding harmonic relationships that could introduce rhythmic overlap. A short beep marked the beginning of a 5-second MI period, followed by a 2-second break to conclude the trial.

The session comprised three runs, each consisting of 35 trials—10 trials for each motor imagery MI task and 5 for the *rest* condition—resulting in a total of 30 trials per MI class and 15 trials for *rest*. Participants were given a break of at least one minute between runs. See Supplementary Materials for experimental setup.

### C. Deep Learning Classification

This study utilizes the EEGConformer model for decoding MI tasks. The model architecture and parameters are depicted in Fig. 3(b). The EEGConformer architecture comprises three fundamental modules: a convolutional module, a self-attention module, and a classification module, designed to classify four MI tasks, including *rest*, *slow*, *medium*, and *fast*. See Supplementary Materials for EEG processing and deep learning training scheme.

### D. Feature Visualization

#### 1) Steady-State Evoked Potential Analysis

EEG signals were analyzed to confirm the presence of SSMRPs and SSVEPs by assessing the power spectral density (PSD) and signal-to-noise ratio (SNR) during the MI period, which are standard approaches for observing steady-state neural responses [59], [60]. Epochs were extracted for two channel sets: occipital and parietal (Pz, O1, and O2) for SSVEP analysis, and frontocentral, central, and centroparietal (FC3, FCz, FC4, C3, Cz, C4, CP3, CPz, and CP4) for SSMRP analysis. The current source density transformation was applied to enhance spatial resolution. See Suplementary Materials for PSD and SNR calculations.

#### 2) Convolutional Layers Interpretation

The interpretation of convolutional layers follows Salami et al. [31]. Temporal convolutions act as frequency filters, where the output $\boldsymbol{Y}[n]$ results from convolving the input $\boldsymbol{X}[n]$ with a time-reversed kernel $\boldsymbol{K}[-n]$. The Fourier transform of each kernel reveals which frequency bands the model prioritizes, and the responses are smoothed using a Savitzky–Golay filter (window = 5, order = 3).

The spatial convolution functions similarly to common spatial patterns [61], projecting temporally filtered features into a source space $\boldsymbol{S}[n]$ using a spatial filter matrix $\mathbf {W}$. To interpret channel relevance, the inverse matrix $\mathbf {W}^{-1}$ is visualized as a spatial pattern, indicating the scalp distribution of learned features.

#### 3) Class Activation Visualization

This study employed two distinct feature visualization techniques for global representation: class activation mapping (CAM) and class activation topography (CAT). The CAM plot uses Gradient-Weighted CAM as described in [30] to highlight the time periods during which the EEGConformer focuses on the EEG features extracted by the convolution module. The CAT plot is generated by multiplying the normalized EEG signals by the CAM to visualize the spatial areas where the model directs its attention, similar to the method described by [28].

Specifically, the CAM plot illustrates the temporal periods during which the model pays the most attention, i.e., when the most informative features are extracted from the EEG signals. In the MI-EEG context, positive CAM values indicate regions or time points associated with ERS that support class prediction, whereas negative CAM values reflect ERD that the model also leverages for discrimination. Similarly, CAT visualizes the spatial distribution of model attention across EEG channels, highlighting the scalp regions most informative for distinguishing each MI task.

## Supplementary Materials

Supplementary Materials

## ETHICS STATEMENT

The experimental protocol was approved by the Ethics Committee of the Graduate School of Engineering, Tohoku University (19A-1). Prior to the experiment, each participant gave informed consent following the Declaration of Helsinki. They were informed that their identifiable data and images may appear in publication but would be anonymized or pseudonymized to protect their privacy and confidentiality.

## CONFLICT OF INTEREST

The authors declare no conflict of interest.

## AUTHOR CONTRIBUTIONS

**ST**: conceptualization, data curation, formal analysis, investigation, methodology and software. **CP**: conceptualization, software, validation, supervision, writing–original draft, writing–review and editing. **KG**: data curation and software. **KK, DO** and **MH**: conceptualization, project administration, resources, methodology, validation, supervision, writing–review and editing.
