# Supplementary material for: Deep Learning-Based Decoding and Feature Visualization of Motor Imagery Speeds From EEG Signals
Source: IEEE Open J Eng Med Biol. 2025 Dec 18;7:27–34. doi: 10.1109/OJEMB.2025.3645617 (PMC12885490; doi:10.1109/OJEMB.2025.3645617)
Supplement: Supplementary Materials [file supp1-3645617.pdf]

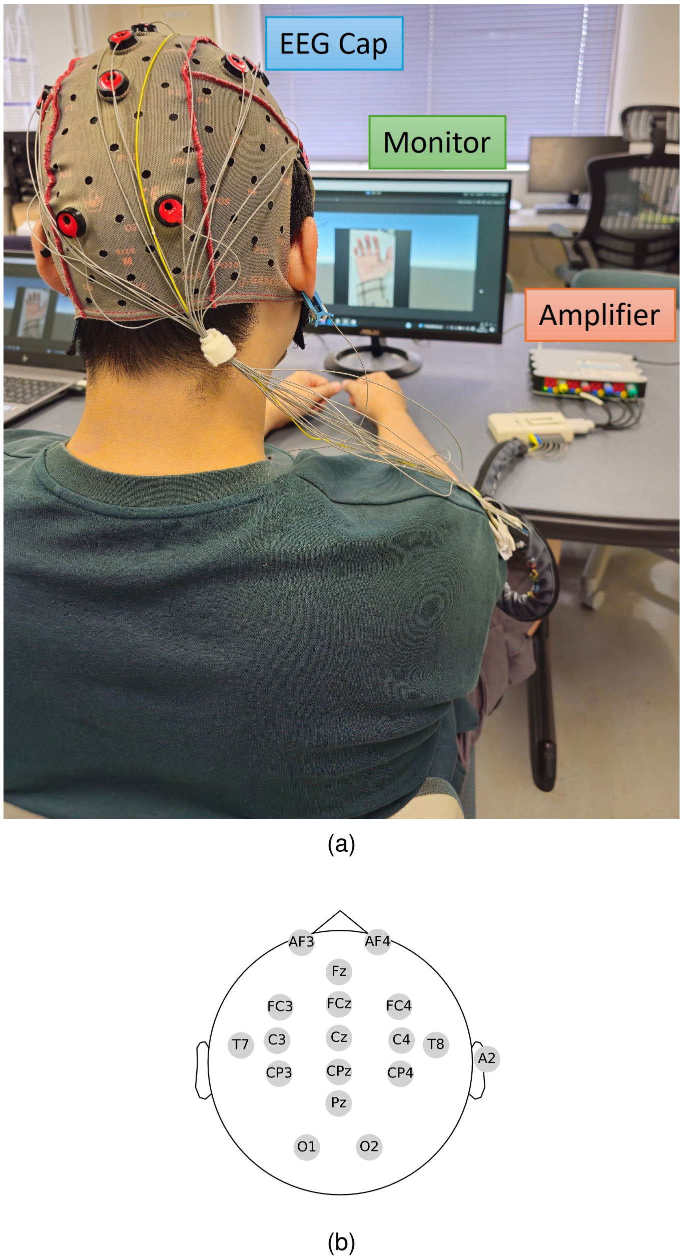

Fig. S1: Overview of the experimental setup: (a) Experimental equipment includes an EEG cap, amplifier, and monitor. (b) Placement of EEG electrodes.

SUPPLEMENTARY MATERIALS

Experimental setup

The experimental setup overview is depicted in Fig. S1. Participants were comfortably seated approximately 1 m from a 24-inch LED monitor as shown in Fig. S1a. EEG data acquisition utilized a 16-channel, 24-bit g.USBamp (g.tec Medical Engineering GMBH) amplifier, operating at a sampling rate of 512 Hz. The amplifier has an input impedance greater than 10 GΩ and a sensitivity of ±250 mV. Active wet Ag/AgCl electrodes were positioned at AF3, AF4, FC3, FCz, FC4, C3, Cz, C4, T7, T8, CP3, CPz, CP4, Pz, O1, and O2. The reference electrode was on the right earlobe (A2), and the ground electrode was at Fz, as shown in Fig. S1b. Participants were instructed to minimize eye movements, head movements, and jaw clenching to avoid artifacts in the recorded EEG data. The EEG signals were filtered using an eighth-order Butterworth band-pass filter (0.5-100 Hz) and a fourth-order Butterworth notch filter (48-52 Hz).

EEG processing for deep learning classification

The EEG signals were further preprocessed following similar steps described in [1]. A third-order Butterworth band-pass filter with cutoff frequencies between 4 and 38 Hz was applied, after which the signals were downsampled to 256 Hz and standardized using Z-scores to reduce noise and mitigate nonstationarity. Each trial was then epoched over the time interval from -1 to 5 s.

Deep learning training scheme

The deep learning framework was implemented utilizing PyTorch 2.1 and executed on an NVIDIA GeForce GTX 2080 GPU. Segmentation and reconstruction method in the time domain was adopted for data augmentation [2]. The training was conducted using the Adam optimizer, with a learning rate of 5×10^{-4}, β_1 set at 0.9, and β_2 set at 0.999. A weight decay of 1×10^{-5} was applied to prevent overfitting. Furthermore, cosine annealing learning rate scheduling was employed to adjust the learning rate dynamically throughout the training process. The batch size for training was set to 64, and the training was carried out for 100 epochs. Cross-entropy was employed as the loss function. These parameters were empirically determined to yield the best results through trial and error. The experiment was conducted using stratified 10-fold cross-validation.

Steady-state evoked potential analysis

Repetitive rhythmic movements are known to induce sensorimotor steady-state response potentials (SSMRPs) [3], characterized by increased EEG activity at the frequency of the movement and its harmonics, particularly over the motor cortex [4]. In this study, participants were also presented with visual cues corresponding to the MI tasks. These visual stimuli can evoke steady-state visually evoked potentials (SSVEPs), which similarly result in enhanced EEG activity at the stimulus frequency and its harmonics, predominantly over parietal and occipital regions. Detecting SSMRPs and SSVEPs is particularly relevant for decoding MI speed, as both reflect frequency-locked neural responses that are closely related to the temporal dynamics of motor execution and visual entrainment. Hence, their presence provides important neural markers for evaluating the extent to which EEG activity responses to the rhythmic properties of MI speed.

EEG signals were analyzed to confirm the presence of SSMRPs and SSVEPs by assessing the power spectral density (PSD) and signal-to-noise ratio (SNR). PSD was computed using Welch's method with a Blackman window and converted to the decibel (dB) scale. SNR was estimated by comparing the power at each frequency to the mean power of neighboring bins (±5 bins, excluding the center). Frequencies at the spectrum edges were excluded.

References

[1] R. T. Schirrmeister, J. T. Springenberg, L. D. J. Fiederer, M. Glasstetter, K. Eggensperger, M. Tangermann, F. Hutter, W. Burgard, and T. Ball, “Deep learning with convolutional neural networks for eeg decoding and visualization,” *Human brain mapping*, vol. 38, no. 11, pp. 5391–5420, 2017.

[2] F. Lotte, “Signal processing approaches to minimize or suppress calibration time in oscillatory activity-based brain–computer interfaces,” *Proceedings of the IEEE*, vol. 103, no. 6, pp. 871–890, 2015.

[3] Y. Wei, X. Wang, R. Luo, X. Mai, S. Li, and J. Meng, “Decoding movement frequencies and limbs based on steady-state movement-related rhythms from noninvasive eeg,” *Journal of Neural Engineering*, vol. 20, no. 6, p. 066019, 2023.

[4] A. M. Norcia, L. G. Appelbaum, J. M. Ales, B. R. Cottereau, and B. Rossion, “The steady-state visual evoked potential in vision research: A review,” *Journal of vision*, vol. 15, no. 6, pp. 4–4, 2015.
